# Lipid nanoparticle-delivered mRNA vaccine encoding the MOMP of *Chlamydia psittaci* elicits protective immune responses in BALB/c mice

**DOI:** 10.1128/spectrum.01438-25

**Published:** 2025-11-05

**Authors:** Buwei Wang, Jian Xiao, Jiewen Wang, Wanying Zhang, Yinqi Jin, Yalan Jiang, Yuqing Chen, Zhangping He, Aihua Lei, Chunxue Lu, Chuan Wang

**Affiliations:** 1 Institute of Pathogenic Biology, School of Basic Medicine, Hengyang Medical College, University of South China 34706, Hengyang, Hunan, China; 2 Department of Laboratory Medicine, Hengyang Medical College, The Affiliated Nanhua Hospital, University of South China 34706, Hengyang, Hunan, China; 3 Clinical Microbiology Laboratory, Xiangtan Central Hospital 117752, Xiangtan, Hunan, China; University of Minnesota Twin Cities, Minneapolis, Minnesota, USA

**Keywords:** *Chlamydia psittaci*, mRNA vaccine, MOMP, lipid nanoparticle, protective immune response

## Abstract

*Chlamydia psittaci* is an important pathogenic zoonotic pathogen that infects birds and can be transmitted to humans and other animals. This research focused on creating an mRNA vaccine targeting *C. psittaci* and assessing its immunogenic and protective effects in BALB/c mice. We carried out a trial to prepare and assess an mRNA vaccine targeting *C. psittaci*. This study involves an mRNA vaccine encoding the major outer membrane protein (MOMP), produced via an *in vitro* transcription system and encapsulated in lipid nanoparticles (LNPs). The mRNA vaccine was constructed by synthesizing non-replicating mRNA encoding MOMP. LNPs were produced and characterized for their particle morphology, size, and cytotoxicity. Histopathological analysis and indirect immunofluorescence assay of lung tissue demonstrated that mRNA vaccines effectively reduce chlamydial infection and shedding in BALB/c mice. The western blot assay confirmed the successful expression of the recombinant MOMP mediated by the target mRNA in HeLa cells. Immunization with the LNP-Opt-mRNA vaccine induced a strong immune response in mice. Mice immunized with the LNP-Opt-mRNA vaccine exhibited lower levels of *C. psittaci* load and decreased concentrations of interferon-γ, TNF-α, and IL-6 in the lungs compared to the PBS group. The study successfully constructed and developed the LNP-mRNA vaccine expressing the MOMP of *C. psittaci*. The vaccine demonstrated strong immunogenicity, inducing effective humoral and cellular immune responses that significantly decreased pulmonary *C. psittaci* burden in mice. These findings provide a basis for designing mRNA vaccines against *C. psittaci* and similar respiratory pathogens.

**IMPORTANCE:**

*Chlamydia psittaci* is a significant threat to public health due to its zoonotic nature and capability to spread from birds to humans and other animals. It is necessary to develop an effective preventive measure. This study introduces an innovative strategy against *C. psittaci* using an mRNA vaccine that encodes the major outer membrane protein, demonstrating potential in eliciting robust immune responses in mice. The work emphasizes the efficacy of mRNA vaccines in inducing humoral and cellular immune responses, significantly decreasing the lung *C. psittaci* burden in mice and underscoring their potential against respiratory pathogens. The study indicates that lipid nanoparticle-mRNA vaccines may be a useful technique for treating and preventing *C. psittaci* infections, providing valuable insights for developing vaccines against other *Chlamydia* species and respiratory pathogens. Finally, this study provides a theoretical foundation and practical experience for the production of *C. psittaci* vaccines, paving the way for further exploration of mRNA vaccine platforms and co-delivery strategies to enhance immune responses.

## INTRODUCTION

Psittacosis is a zoonotic illness caused by *Chlamydia psittaci*, an obligate intracellular pathogen from the Chlamydiales order. It is characterized by a distinctive biphasic developmental cycle that includes the differentiation of elementary and reticulate bodies. This pathogen demonstrates strict tropism for eukaryotic host cells, enabling the induction of diverse pathological manifestations. Avian infections typically present with pulmonary complications (e.g., respiratory distress), reproductive disorders (abortion and stillbirth), and systemic dissemination. Human psittacosis manifests as severe pulmonary involvement, extrapulmonary complications, including septic shock, neurological manifestations (e.g., meningitis and cranial nerve paralysis), and cardiovascular involvement (myocarditis and pericarditis) (1–5). Recently, with the continuous development of clinical diagnostic techniques, the incidence of *C. psittaci* has been increasing, indicating that *Chlamydia* infection may be greatly underestimated (6).

Furthermore, Chlamydial infections frequently manifest as asymptomatic or subclinical presentations, and empirical antibiotic therapy may precipitate recrudescent infection (7, 8). This dual burden of public health risk and compromised productivity in critical agricultural sectors (e.g., poultry and livestock) underscores the necessity of *Chlamydia* vaccine development. Successful implementation of such prophylactic strategies could mitigate zoonotic transmission dynamics. A lot of researchers have exerted efforts in this domain (9–12). Recent advancements have established mRNA as a promising platform for vaccines. Modified nucleosides like pseudouridine and N-1-methylpseudouridine significantly enhance protein production *in vivo* (13, 14). Post-transcriptional chemical modifications, such as 2′-O-methylation that establishes a canonical Cap 1 structure on synthetic mRNA, have been shown to improve ribosomal engagement and significantly reduce nonproductive innate immune responses by mimicking endogenous eukaryotic mRNA structures. Consequently, vaccine technology is at a pivotal stage, with mRNA vaccines undergoing clinical trials for diseases like HIV, influenza, Zika, and various cancers (15–18). This study aims to evaluate the effectiveness of a *C. psittaci* mRNA vaccine in preventing *C. psittaci* infection.

mRNA vaccines function by introducing mRNA sequences that encode antigen proteins into host cells, where the host cell’s expression system synthesizes these proteins. This process initiates an immune response targeting the specific protein in the host, with the objective of preventing disease (19). Antigen selection is vital for developing an optimal *Chlamydia* mRNA vaccine due to its direct impact on the vaccine’s immunogenicity, efficacy, and safety (20). The major outer membrane protein (MOMP) constitutes approximately 60% of the total outer membrane proteins in *Chlamydia*. It is essential for the integrity of the outer membrane structure, growth metabolism, antigenicity, and virulence (21). However, the inability of native MOMP to induce cross-seroprotection and the fact that exogenously expressed MOMP (referred to as recombinant MOMP, rMOMP) lacks a natural conformation have limited the applicability of MOMP (22–29). The difficulties in using MOMP as a vaccine antigen prompted us to investigate the potential of an mRNA vaccine for MOMP. This project aims to first develop a *C. psittaci* mRNA vaccine based on MOMP, providing theoretical and experimental foundations for *Chlamydia* vaccine research.

## MATERIALS AND METHODS

### Construction and identification of the pET-28a(+)/MOMP plasmid

The *MOMP* gene (GenBank: X56980.1) was amplified from the genomic DNA of *C. psittaci* 6BC (Ref Seq: NC_017288) utilizing specific primers (forward:5′-CGCGGATCCATGAAAAAACTCT-3′; reverse:5′-CCGCTCGAGTTAGAATCTG-3′) and Phanta Max Super-Fidelity DNA Polymerase (Vazyme, China). The amplified product was inserted into pET-28a(+) (Sangon Biotech, China) using *BamHI*/*XhoI* restriction sites (the underlined part is the enzyme digestion site), confirmed through PCR, electrophoresis, and sequencing, and purified with the E.Z.N.A. Cycle-Pure Kit (Omega, USA) and stored at -20°C.

### Expression and purification of MOMP

The pET-28a(+)/MOMP plasmid was introduced into *Escherichia coli* BL21(DE3) (Sangon Biotech, China) and induced with 0.5 mM IPTG at an OD_600_ of 0.8 under conditions of 16°C and 180 rpm for 6 h. The expressed protein was purified via ultrasonication, Ni-NTA affinity chromatography (Cytiva, USA), and endotoxin removal (Beyotime, China), followed by SDS-PAGE validation. Protein concentration was measured using the BCA assay (Epizyme, China). The purified rMOMP was used for subsequent detection of MOMP-specific antibody titers in mouse serum and served as a control for Western blot analysis.

### Plasmid engineering for *C. psittaci* MOMP mRNA design

We engineered expression plasmids by incorporating the full-length *C. psittaci* 6BC (ATCC VR-125) MOMP gene (Fig. S1A). In this study, human β-globin was attached to the untranslated region (UTR) to enhance mRNA stability and translation efficiency (30, 31): 5′ UTR from human β-globin-2 and the 3′ UTR from human 2β-globin (Fig. S1B). These genetic constructs were cloned into the pGEM‐3Zf(+) vector (Ke Lei Biological Technology Co., Ltd, China) (32–34). This is called pGEM_UTRs_. The coding sequence of *C. psittaci* 6BC *MOMP* gene was inserted by molecular cloning technology to construct the recombinant plasmid vector pGEM_MOMP_ using *AscI*/*PacI* restriction sites (the underlined part is the enzyme digestion site). It utilized specific primers (forward:5′-AGGCGCGCCATGAAAAAACTCTTGA-3′; reverse:5′-CCTTAATTAATTAGAATCTGAA-3′). All recombinant plasmids were isolated and purified using the Endo-Free Plasmid Maxi Kit (Omega, USA). The enzyme products were identified by 0.5% agarose gel electrophoresis, and the results were observed and stored under the gel imaging system. The purified plasmids were subsequently stored at -20°C to maintain integrity until further use.

### mRNA transcription *in vitro*

Linearized pGEM_UTRs_ and pGEM_MOMP_, prepared with *BamHI* (NEB, USA), served as DNA templates for IVT and were purified using the phenol-chloroform method. Similarly, the purified product was identified by 0.5% agarose gel electrophoresis. The purified linearized recombinant plasmids, pGEM_UTRs_ and pGEM_MOMP_, underwent *in vitro* transcription with the HiScribe T7 Quick High Yield RNA Synthesis Kit (NEB, USA), incorporating capping reactions during the process. The mRNA products underwent a tailing reaction using the *E. coli* Poly(A) Polymerase kit (NEB, USA), resulting in capped and tailed mRNA sequences. These sequences were designated as mRNA_UTRs_ and mRNA_MOMP_, respectively.

Opt-mRNA_MOMP_ was synthesized using the HyperScribe All in One mRNA Synthesis Kit Plus 1 (APExBIO Technology LLC, USA) with T7 RNA polymerase from linearized pGEM_MOMP_. The optimized construct included an anti-reverse 3′-O-Me-m7G(5′)ppp(5′)G cap analog, along with 5mCTP, and ψUTP modifications to enhance stability, optimize translation efficiency, and reduce immunogenicity. The concentration and purity of the synthesized mRNA were assayed via absorbance at 260 nm, using the spectrophotometer (DenoVIX, USA). The mRNA was isolated using phenol-chloroform extraction and preserved at -80°C.

### mRNA transfection

HeLa 229 cells (ATCC CCL-2.1) were cultured in 6-well plates (1 × 10⁶ cells/well) using Dulbecco’s Modified Eagle Medium (DMEM; Gibco, USA) supplemented with 10% fetal bovine serum (DMEM-10), maintained at 37°C with 5% CO_₂_ until reaching 80% confluency. Following PBS washing, each well received 500 µL of Opti-MEM medium. Lipofectamine 3000 (Thermo Fisher Scientific, USA) was used for transfection with 2.5 µg of mRNA per well. After incubating at 37°C with 5% CO_₂_, 500 µL of DMEM-10 was added at 6 h. Protein extraction was performed 48 h after transfection.

### Western blot analysis

Following transfection, protein expression in HeLa cells treated with mRNA_UTRs_, mRNA_MOMP_, and Opt-mRNA_MOMP_, along with a PBS control, was assessed using Western blot analysis. Proteins were meticulously separated on a 12.5% SDS-PAGE gel and electro-transferred onto 0.22 µm PVDF membranes. The membranes were blocked in a 5% non-fat dry milk solution at 37°C for 2 h. Subsequently, the membranes were incubated overnight at 4°C with a 1:500 dilution of rabbit anti-*C*. *psittaci* polyclonal antibody provided by Dr. Zhong from the University of Texas Health Science Center. After washing, blots were probed with HRP-linked goat anti-rabbit IgG (1:2,000, Abcam) for 1 h. Protein signals were detected using a high-sensitivity chemiluminescence substrate (Epizyme Biotech, China).

### Preparation and identification of LNP and LNP-mRNA

The formulation and preparation method of LNP-mRNA followed the approach previously described in references 32, 35–37. LNPs were prepared using a molar ratio of 50:50:1 for DOTAP, DOPE, and DSPE-PEG2000 (MedChemExpress, USA). For LNP assembly, an ethanol-dissolved lipid mixture and aqueous mRNA solution were combined at a 3:1 volumetric ratio using a microfluidic device (Precision NanoSystems). This process promotes the instantaneous self-assembly of lipids around the mRNA strands, forming complete LNP-mRNA particles. The resulting LNP-mRNA complexes were concentrated using centrifugal ultrafiltration (10 kDa cutoff, Millipore, USA). The size distribution, zeta potential, and polydispersity index of LNPs and LNP-mRNA complexes were determined by dynamic light scattering using a Malvern Zetasizer Nano ZS90. Morphological examination was performed using transmission electron microscopy (TEM). For TEM imaging, the LNPs were appropriately diluted and applied onto a carbon-coated copper grid. After 1 min of adsorption, the excess liquid was blotted away with filter paper. The grid was then negatively stained with 2% phosphotungstic acid (pH 7.0) for 1 min, followed by blotting to remove the excess stain. The grid was air-dried thoroughly at room temperature prior to observation under the transmission electron microscope. To evaluate the binding affinity between LNPs and mRNA, an agarose gel retardation assay was conducted. Appropriate amounts of LNP-mRNA complexes were electrophoresed on a 1.2% agarose gel at 100 V for 45 min. After electrophoresis, the gel was carefully imaged using a gel documentation system for analysis.

### Cytotoxicity assay of LNPs

The cytocompatibility of LNPs across different N/P ratios was assessed using CCK-8 assays (Bimake, USA) to define their optimal therapeutic window while reducing cytotoxicity. According to the kit instructions, cellular metabolic activity was assessed after 24 h of exposure under standard culture conditions by measuring the reduction of WST-8 to formazan at 450 nm following 4-h incubation with CCK-8 reagent.

### Transfection and identification of LNP-mRNAs into HeLa cells

HeLa cells were seeded in 6-well plates in DMEM-10. Gene delivery was performed using engineered LNP formulations, with 5 µg of LNP-mRNA_MOMP_ or its codon-optimized variant LNP-Opt-mRNA_MOMP_. Parallel control groups included LNP-mRNA_UTRs_ and PBS. Post-transfection recombinant protein expression was quantitatively assessed 24 h post-treatment using immunoblotting to determine transfection efficiency.

### Immunogenicity studies

Forty 6-week-old SPF female BALB/c mice from Hunan SJA Laboratory Animal Co., Ltd, China, were organized into four groups. No additional adjuvant was used, as the LNP delivery system itself provides potent adjuvant activity. The following groups were included in the study: an experimental group that received LNP-mRNA_MOMP_, an optimized group that was treated with LNP-Opt-mRNA_MOMP_, a negative control group administered LNP-mRNA_UTRs_, and a PBS blank control group. Each mouse underwent a series of three intramuscular immunizations, administered biweekly, with the objective of establishing a robust immune response. The doses were meticulously calibrated, with each mouse receiving 15 µg of the respective mRNA formulation or PBS, diluted in 100 µL of sterile PBS to ensure consistent delivery.

Fourteen days following the final immunization, 20 of them were euthanized, and their spleens were aseptically collected for flow cytometry and lymphocyte proliferation assays to evaluate vaccine-induced cellular immunity. A series of blood samples was collected at strategic intervals of 14, 28, and 42 days following the initial immunization, with the objective of monitoring the production of antibodies. Thereafter, serum was stored at a temperature of -80°C.

### Preparation of *C. psittaci* and intranasal challenge

The propagation of *C. psittaci* was conducted as follows: McCoy cell monolayer (thanks to Prof. Chunfu Yang of SUSTech for the provision of McCoy cells) was inoculated with *C. psittaci* via centrifugation-assisted infection (545 × *g*, 1 h, and 37°C), followed by incubation in DMEM-10, 1 µg/mL gentamicin, and 1 µg/mL cycloheximide. Following the process, *C. psittaci* were resuspended in 100 µL of SPG buffer (10 mM sodium phosphate, 0.25 M sucrose, and 5 mM L-glutamic acid, pH 7.2). Aliquots were cryopreserved at -80°C for the purpose of long-term storage.

The remaining 20 mice (*n* = 5/group) were intranasally challenged with *C. psittaci* (5 × 10^5^ inclusion-forming units) in 30 µL SPG buffer. On the 10th day following infection, all mice were humanely euthanized, and lungs were aseptically obtained for quantitative assessment of the immunoprotective efficacy against infection.

The chlamydial inclusion burden was determined by microscopic enumeration of inclusion-forming units (IFUs) following tissue homogenization, serial dilution, and immunofluorescence staining, with final counts normalized.

### Flow cytometry

The staining solution was prepared in the dark and included 0.2 µL each of FITC-CD3ε, PerCP-CD4, and APC-CD8a antibodies for mice. Next, 50 µL of PBS was added to the mixture and mixed thoroughly. Then, 5 µL of pre-treated splenocyte suspension was transferred to a flow tube. Each tube was then supplemented with 50 µL of the staining solution and thoroughly mixed with the sample. The reaction was incubated at 4°C. Finally, 200 µL of PBS was added to each tube to terminate the reaction and ensure that the experiment could be conducted within 12 h.

### Spleen lymphocyte proliferation assay

Splenocyte suspensions were quantified via Neubauer chamber counting and adjusted to 2 × 10^6^ cells/mL in sterile RPMI-1640 medium. Aliquots (100 µL/well) were plated in triplicate into 96-well plates under three conditions: MOMP-stimulated (10 µg/mL MOMP), unstimulated, and medium-only blank controls. After a 48-h incubation (37°C and 5% CO_2_), 10 µL of CCK-8 reagent was added to each well. After 1–4 h dark culture, the optical density at 450 nm was measured using a microplate reader.

### Indirect immunofluorescence assay

To further refine the indirect immunofluorescence assay (IFA) for detecting *C. psittaci* inclusions, this study introduces several optimizations to enhance the sensitivity and accuracy of the visualization process. McCoy cells were cultured in DMEM medium designed to support higher survival rates post-infection.

At 42 h post-infection, McCoy cells were fixed with 4% formaldehyde and permeabilized using 0.1% Triton X-100 to enhance antibody penetration and staining intensity.

### Quantification of serum MOMP-specific IgG titers via indirect ELISA

Recombinant MOMP, purified and devoid of endotoxins, was adsorbed onto 96-well plates at a concentration of 10 µg/mL in carbonate buffer (pH 9.6), using 100 µL per well, and incubated overnight at 4°C. After three washes with PBST (3×, 200 µL, 3 min each), plates were blocked using 5% nonfat milk-PBST (200 µL per well) for 2 h at 37°C. Mouse serum was serially diluted in a 5% milk-PBST solution, and 100 µL was added per well. The samples were incubated for 2 h at 37°C and then washed with PBST. HRP-conjugated goat anti-mouse IgG was diluted 1:8,000 in 5% milk-PBST, and 100 µL was added per well, incubating for 2 h at 37°C. After final PBST washes, TMB substrate (100 µL/well) was incubated (15 min, 37°C, dark), and reactions were terminated.

### Detection of cytokine levels in lungs

To elucidate the immunological response to *C. psittaci* infection, the levels of IFN-γ, TNF-α, IL-2, IL-4, IL-6, and IL-10 were measured utilizing high-sensitivity ELISA kits (eBioscience, USA). Adjustments to the ELISA protocol included optimized antibody concentrations and advanced signal amplification, ensuring precise and reproducible cytokine measurements.

### Histopathology

Lung specimens were preserved using 4% paraformaldehyde, infiltrated in paraffin wax, consecutively cut into thin slices, and treated with hematoxylin and eosin (H&E) dye for analysis.

### Quantitative PCR

Genomic DNA was isolated from homogenized murine pulmonary specimens using the TIANamp Genomic DNA Kit (TIANGEN Biotech, China), with subsequent quantification of *C. psittaci* load performed via quantitative real-time PCR. The primer sequences are as follows:

- 16s rRNA-Forward:5′-TCCGCAAGGACAGATACACA-3′
- 16s rRNA-Reverse:5′-ACCCAGGCAGTCTCGTTAGA-3′
- β-actin-Forward:5′-CCTTCCTTCTTGGGTATGGA-3′
- β-actin-Reverse:5′-ACGGATGTCAACGTCACACT-3′

The chlamydial inclusion burden was determined by microscopic enumeration of IFA following tissue homogenization, serial dilution, and immunofluorescence staining, with final counts normalized.

### Statistical analysis

All measurements in every group were expressed with mean ± SD and analyzed using Student’s *t*-test, one-way or two-way analysis of variance (ANOVA). All statistical analyses were performed using GraphPad Prism (version 8.0.2, GraphPad Software). A statistically significant threshold was defined as *P*-value < 0.05 for all experimental outcomes.

## RESULTS

### Identification and protein expression of the recombinant prokaryotic expression vector pET-28a(+)/MOMP

**Fig 1:**
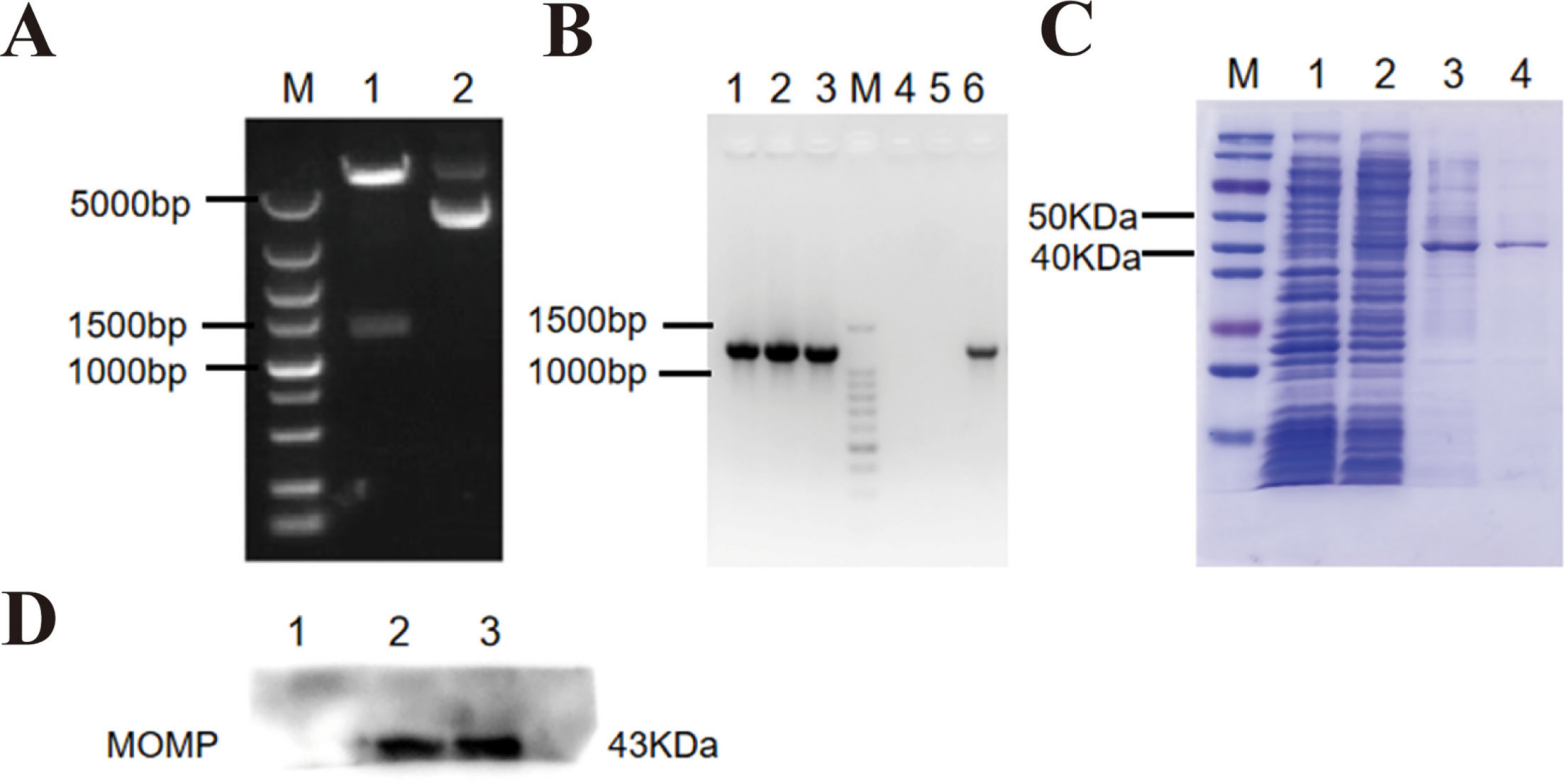
Identification of prokaryotic expression vector pET-28a(+)/MOMP and the protein expression. (**A**) Verification of the vector pET-28a(+)/MOMP by double-enzyme digestion. Lane M: DNA marker; lane 1: pET-28a(+)/MOMP digested by with *BamHI* and *XhoI* restriction enzymes; lane 2: pET-28a(+)/MOMP without restriction digestion. (**B**) Verification of the vector pET-28a(+)/MOMP by bacterial colony PCR. Lanes 1–3: *E. coli* colony with plasmid pET-28a(+)/MOMP; lane M: DNA marker; lanes 4 and 5: *E. coli* colony with empty vector pET-28a(+); lane 6: *C. psittaci* DNA. (**C**) Identification and purification of rMOMP by SDS-PAGE. Lane M: multicolor prestained protein ladder; lane 1: *E. coli* BL21 (DE3) with plasmid pET-28a(+)/MOMP not induced by 0.5 mM IPTG; lane 2: *E. coli* BL21 (DE3) with plasmid pET-28a(+)**/**MOMP induced by 0.5 mM IPTG; lane 3: elution buffer with imidazole (100 mM, 5 mL/elution); lane 4: elution buffer with imidazole (150 mM, 5 mL/elution). (**D**) Identification of rMOMP by Western blot. Lane 1: total protein of *E. coli* BL21 (DE3) with empty vector pET-28a(+) induced by 0.5 mM IPTG; lanes 2 and 3: purified rMOMP detected using rabbit anti-*C*. *psittaci* 6BC strain.

The recombinant vector pET-28a(+)/MOMP was analyzed using *BamHI* and *XhoI* double digestion and agarose gel electrophoresis. Figure 1A illustrates that the digested products produced fragments of about 5,369 bp for the empty vector and 1,209 bp for the *MOMP* target gene, aligning with the anticipated sizes. PCR amplification utilized *C. psittaci* 6BC DNA as both a template and positive control, alongside colonies containing either the empty vector pET-28a(+) or the recombinant vector pET-28a(+)/MOMP. Results (Fig. 1B) revealed no bands in the pET-28a(+) group, whereas distinct bands matching the size of *C. psittaci* 6BC DNA were observed in the pET-28a(+)/MOMP group, confirming successful construction of the recombinant plasmid. SDS-PAGE analysis was conducted to evaluate rMOMP expression. Figure 1C shows a distinct 43 kDa band in lysates of *E. coli* transformed with pET-28a(+)/MOMP following induction with 0.5 mM IPTG, which is absent in uninduced (0 mM IPTG) conditions. Following purification, the target protein was efficiently eluted in buffer containing 150 mM imidazole, with minimal contaminant bands observed. Western blot analysis further validated the identity of the purified rMOMP. Using total protein lysates from *E. coli* with the empty vector pET-28a(+) as a negative control, specific 43 kDa bands were identified in the rMOMP group when probed with rabbit anti-*C*. *psittaci* 6BC antibody (1:500) (Fig. 1D). Conversely, the negative control group showed no distinct signals. These findings together validate the effective expression, purification, and antigenic specificity of the rMOMP.

### Construction of pGEM_MOMP_

**Fig 2:**
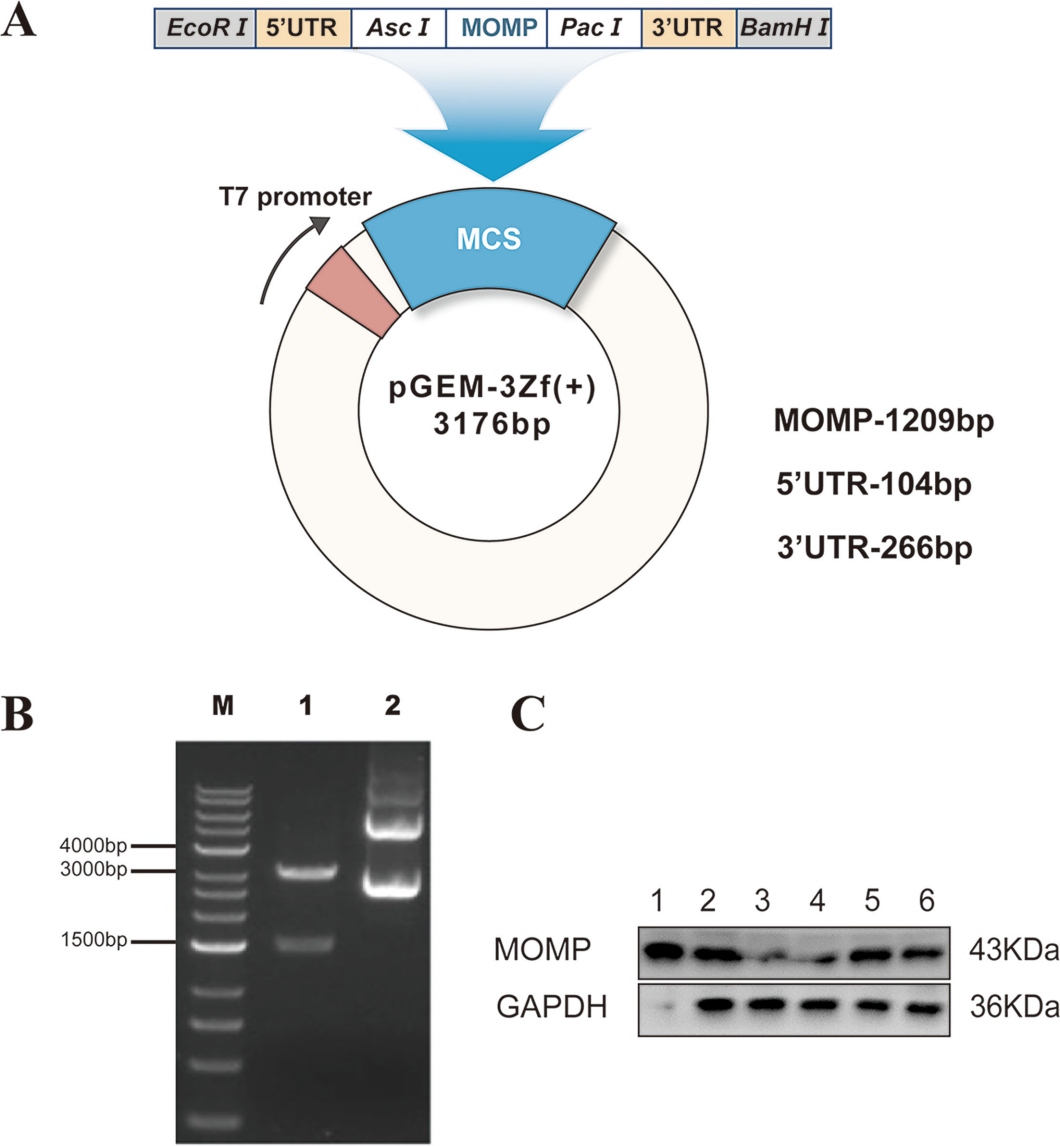
Construction and identification of pGEM_MOMP_ and Opt-mRNA_MOMP_. (**A**) The structural diagram of the recombinant pGEM_MOMP_. (**B**) Double-enzyme digestion of the recombinant pGEM_MOMP_. Lane M: DNA marker; lane 1: pGEM_MOMP_ digested by the *EcoRI* and *BamHI* restriction enzymes; lane 2: pGEM_MOMP_ without restriction digestion. (**C**) Western blot. Lane 1: MOMP protein expressed in *E. coli* BL21(DE3); lane 2: Opt-mRNA_MOMP_ group; lane 3: PBS group (blank control); lane 4: LNP-mRNA_UTR_ group (negative control); lane 5: LNP-mRNA_MOMP_ group; and lane 6: LNP-Opt-mRNA_MOMP_ group (optimized group). The primary antibody used was rabbit anti-*C*. *psittaci* 6BC antibody. GAPDH was the reference standard.

Briefly, pGEM_MOMP_ has the target genes, including *C. psittaci* 6BC *MOMP* gene (1,209 bp), 5′ UTR (104 bp), and 3′ UTR (266 bp), which were inserted into empty pGEM-3Zf(+) vector (3,176 bp) (Fig. 2A). By utilizing the restriction enzymes *EcoRI/BamHI*, the pGEM_MOMP_ was subjected to double enzyme digestion. Subsequently, DNA agarose gel electrophoresis was performed, as illustrated in Fig. 2B. The resulting fragments from the double digestion exhibited an expected band size of approximately 3,176 bp for the empty vector fragment and 1,591 bp for the target gene fragment. This correspondence between the observed band sizes and the expected sizes indicates the successful construction of the pGEM_MOMP_.

We used Western blot analysis to evaluate the biological functionality of Opt-mRNA_MOMP_ target sequences in HeLa cells following IVT by examining MOMP expression. The results are depicted in Fig. 2C. The use of a polyclonal antibody (rabbit anti-*C*. *psittaci* 6BC) resulted in the position of the bands overlapping with that of β-actin. As a result, both the PBS group and the LNP-mRNA_UTRs_ group displayed lighter MOMP bands. The expression intensity of MOMP in the Opt-mRNA_MOMP_, LNP-mRNA_MOMP_, and LNP-Opt-mRNA_MOMP_ groups was higher compared to the LNP-mRNA_UTRs_ and PBS groups, which is consistent with our initial expectations.

### Preparation of LNP and LNP-mRNA_MOMP_

**Fig 3:**
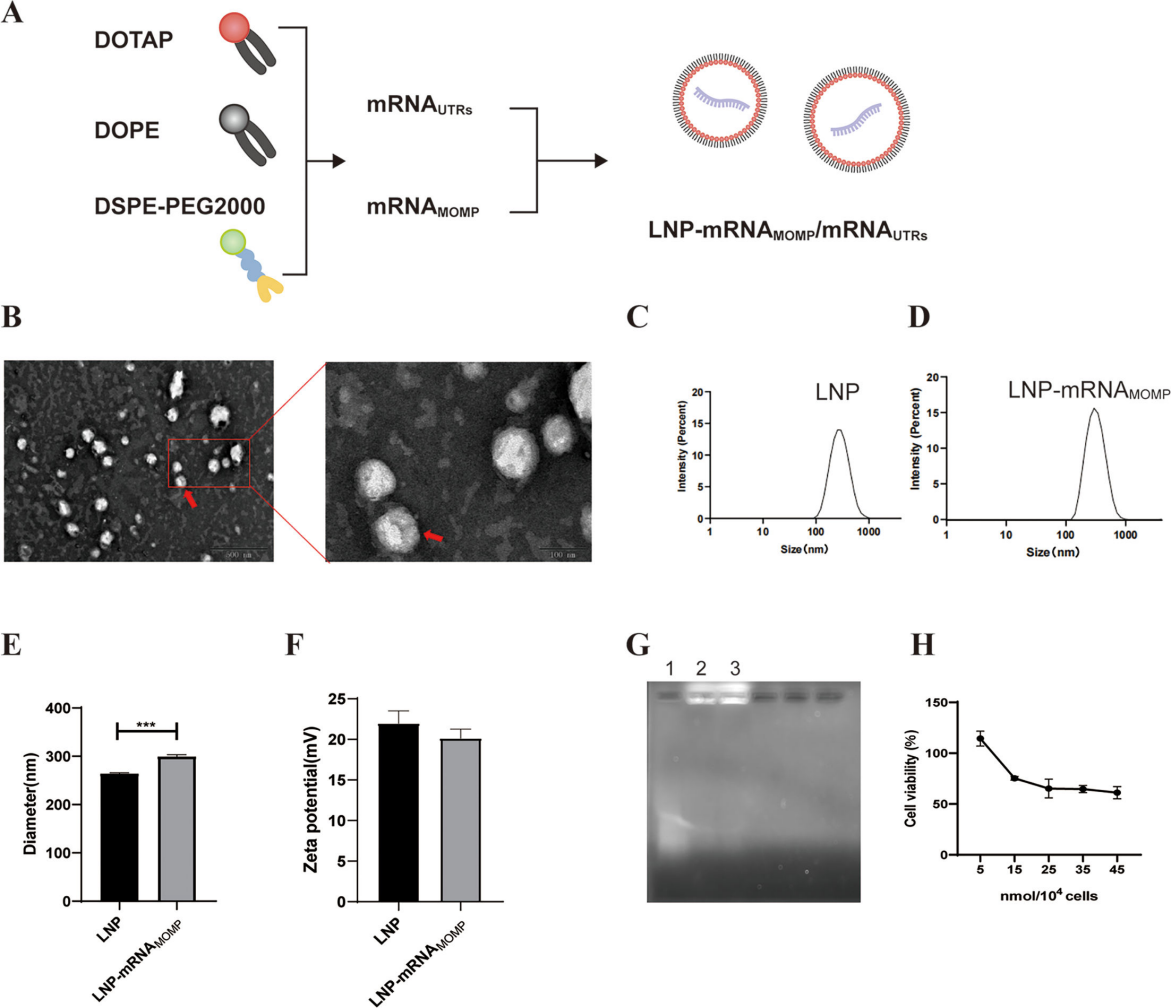
Preparation and identification of LNP and LNP-mRNA_MOMP_. (**A**) Flow chart of LNP and LNP-mRNA_MOMP_ preparation. (**B**) Representative transmission electron microscopy image of LNPs negatively stained with phosphotungstic acid. The predominant, well-defined spherical particles (indicated by red arrows) are the LNPs. Minor background material may include stain precipitate or buffer salts. Intensity (**C and D**), diameter size (**E**), and zeta potential of LNP and LNP-mRNA_MOMP_ (**F**). (**G**) The result of the gel retardation experiment. Lane 1: naked mRNA_MOMP_, not mixed with LNP, was used as the negative control. Lanes 2 and 3: LNP-mRNA_MOMP_. (**H**) Cytotoxicity assay of LNP. Data are shown as mean ± SD and were analyzed by the Student’s *t*-test (***P < 0.001).

A detailed preparation flow chart is given in Fig. 3A. Transmission electron microscopy was used to examine the morphology of the LNP. The findings, illustrated in Fig. 3B, reveal the presence of spherical particles, indicated by the red arrows. To assess the particle size, surface potential, and dispersity of the LNP and its complex with mRNA, the Malvern Zetasizer Nano ZS90 analyzer was utilized. The polydispersity index values for LNP and LNP-mRNA_MOMP_ were approximately 0.169 and 0.132, respectively. These values indicate that the particle size distribution is relatively uniform for both samples, with similar sizes within each group (Fig. 3C and D). The average particle diameter of LNP was about 264.1 nm, whereas LNP-mRNA_MOMP_ had a significantly larger average diameter of approximately 299.5 nm. This increase in particle size after mRNA binding is in accordance with expectations (Fig. 3E). Since one of the components of LNP is the cationic DOTAP, the zeta potential should be positive. Experimental analysis revealed that the surface potential of LNP was approximately 21.97 mV, confirming the positive value as expected. As mRNA carries a negative charge, its zeta potential should be negative. The measured surface potential of LNP-mRNA_MOMP_ was approximately 20.13 mV, slightly lower than that of LNP alone, indicating the electrostatic interaction between LNP and mRNA (Fig. 3F).

The consistent increase in particle size, the attenuation of surface charge, and the low polydispersity collectively suggested the successful formation of LNP-mRNA_MOMP_. To further confirm that the mRNA was completely encapsulated and not merely adsorbed onto the surface, we performed a gel retardation assay. As shown in Fig. 3G, during agarose gel electrophoresis, the bands corresponding to mRNA_MOMP_ without LNP gradually migrated, and the bands appeared more dispersed. In contrast, LNP-mRNA_MOMP_, which were mixed with LNP, remained completely immobilized in the loading well. This observation indicates a close binding between LNP and mRNA_MOMP_, as LNP effectively retards the migration of mRNA_MOMP_.

Determining the safe and effective dosage range of LNP using the CCK-8 assay kit is crucial for advancing *in vitro* transfection experiments with HeLa cells. This will help avoid excessive LNP toxicity, resulting in cell damage. These results, as depicted in Fig. 3H, demonstrate that when the concentration of LNPs is below 15 nmol/10^4^ cells, the cell viability is higher than 75.29%. However, as the concentration of LNP increases, the cell viability shows a decreasing trend.

### Specific antibody response in BALB/c mice

**Fig 4:**
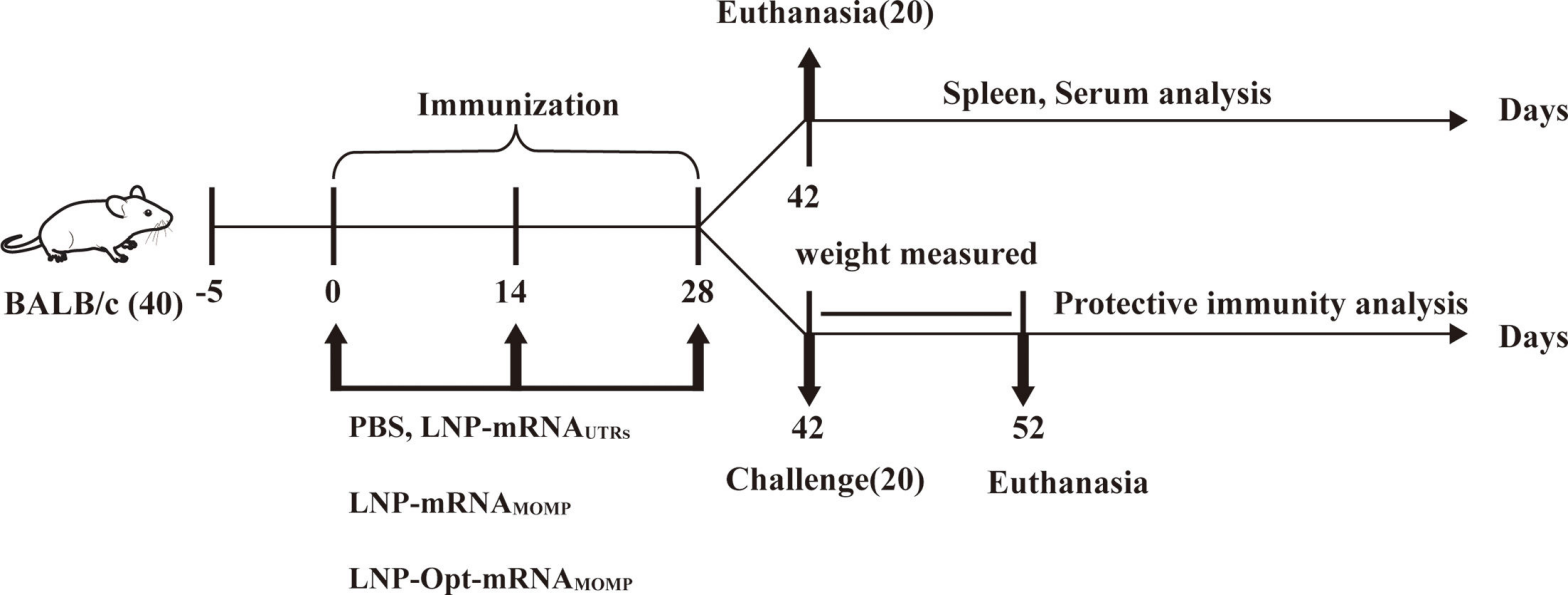
Graphical representation of the murine vaccination protocol. Forty 6-week-old BALB/c female mice were split into four groups (10 mice per group) at random: PBS, LNP-mRNA_UTRs_ group, LNP-mRNA_MOMP_ group, and LNP-Opt-mRNA_MOMP_ group. All groups were immunized by intramuscular injection three times with a 2-week interval between each immunization. Before each immunization, the levels of anti-mouse MOMP-specific serum IgG were detected by ELISA in order to evaluate the vaccine-induced humoral response. Two weeks after the final immunization, half of the mice per group were sacrificed, and the spleen tissue was isolated for lymphocytes proliferation assay and T lymphocyte subset identification. Then, the remaining mice were intranasally infected with 5 × 10^5^ IFUs of *C. psittaci*. Mice were sacrificed on day 10 post-infection, then the cytokine levels in lungs were evaluated by ELISA, and the *C. psittaci* loads were detected.

**Fig 5:**
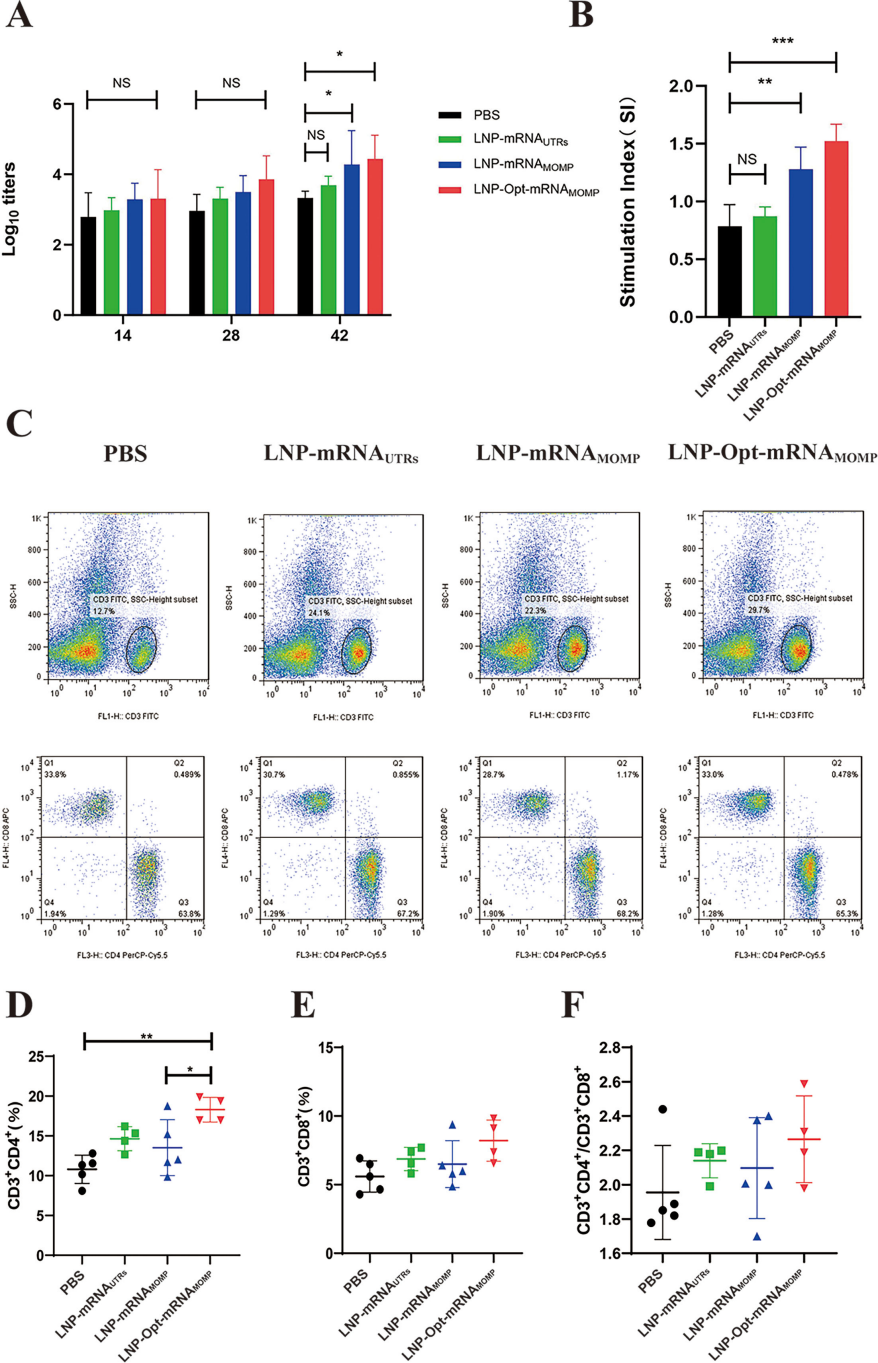
Vaccine-induced antigen-specific humoral immunity, splenic lymphocyte proliferative responses in BALB/c mice, and flow cytometric profiling of T-cell subpopulations. (**A**) Detection of the specific IgG induced by vaccines. (**B**) Stimulation index (SI) of splenic lymphocyte proliferation in mice. SI = (treated group OD − blank group OD)/(untreated group OD − blank group OD). (**C**) Representative results of the flow cytometric determination. (**D**) Results of the CD3^+^CD4^+^ T cells. (**E**) CD3^+^CD8^+^ T cell level. (**F**) The specific value of CD3^+^CD4^+^/CD3^+^CD8^+^ T cells. Data in each group were analyzed by one-way ANOVA test; (NS, no significance; *P < 0.05; **P < 0.01; and ***P < 0.001).

A detailed flow chart of the mouse experiments is shown in Fig. 4. To assess the level of humoral immunity induced by the vaccines in each group, specific IgG antibody levels were detected using ELISA in serum samples collected from mice in each group 14 days after each immunization. The results are illustrated in Fig. 5A, which shows that serum collected 14 and 28 days post-first immunization did not significantly differ between the four groups. Forty-two days post-first immunization, serum analysis showed no significant difference between the LNP-mRNA_UTRs_ group and the PBS group. However, significant differences compared to the PBS group were observed with the LNP-mRNA_MOMP_ group and the LNP-Opt-mRNA_MOMP_ group.

### The LNP-Opt-mRNA_MOMP_ vaccine effectively induces a CD4^+^ T lymphocyte immune response and demonstrates robust T lymphocyte proliferation in BALB/c mice

As an important parameter for the evaluation of the level of specific cellular immune response, we used the CCK-8 method to analyze the proliferation of T lymphocytes in splenic tissue. Figure 5B presents the results. Compared with the stimulation index of the PBS group, the LNP-mRNA_UTRs_ group is not significantly different, while the LNP-mRNA_MOMP_ and LNP-Opt-mRNA_MOMP_ groups were significantly different. The findings indicated that both LNP-mRNA_MOMP_ and LNP-Opt-mRNA_MOMP_ groups exhibited enhanced T lymphocyte proliferation.

Following the euthanasia of the mice, spleen tissues were aseptically extracted, and flow cytometry was utilized to delineate the T lymphocyte subpopulations (Fig. 5C). The LNP-Opt-mRNA_MOMP_ group showed a notable difference in CD3^+^CD4^+^ T lymphocyte levels compared to the PBS group (Fig. 5D). No significant differences were found in CD3^+^CD8^+^ T lymphocyte levels (Fig. 5E) and the CD3^+^CD4^+^/CD3^+^CD8^+^ T lymphocyte subpopulation ratio (Fig. 5F) among the remaining groups when compared to the PBS group. The findings indicate that the LNP-Opt-mRNA_MOMP_ group may predominantly stimulate a CD4^+^ T lymphocyte immune response.

### The LNP-Opt-mRNA_MOMP_ group effectively decreased *C. psittaci* load in the lung tissues of BALB/c mice

**Fig 6:**
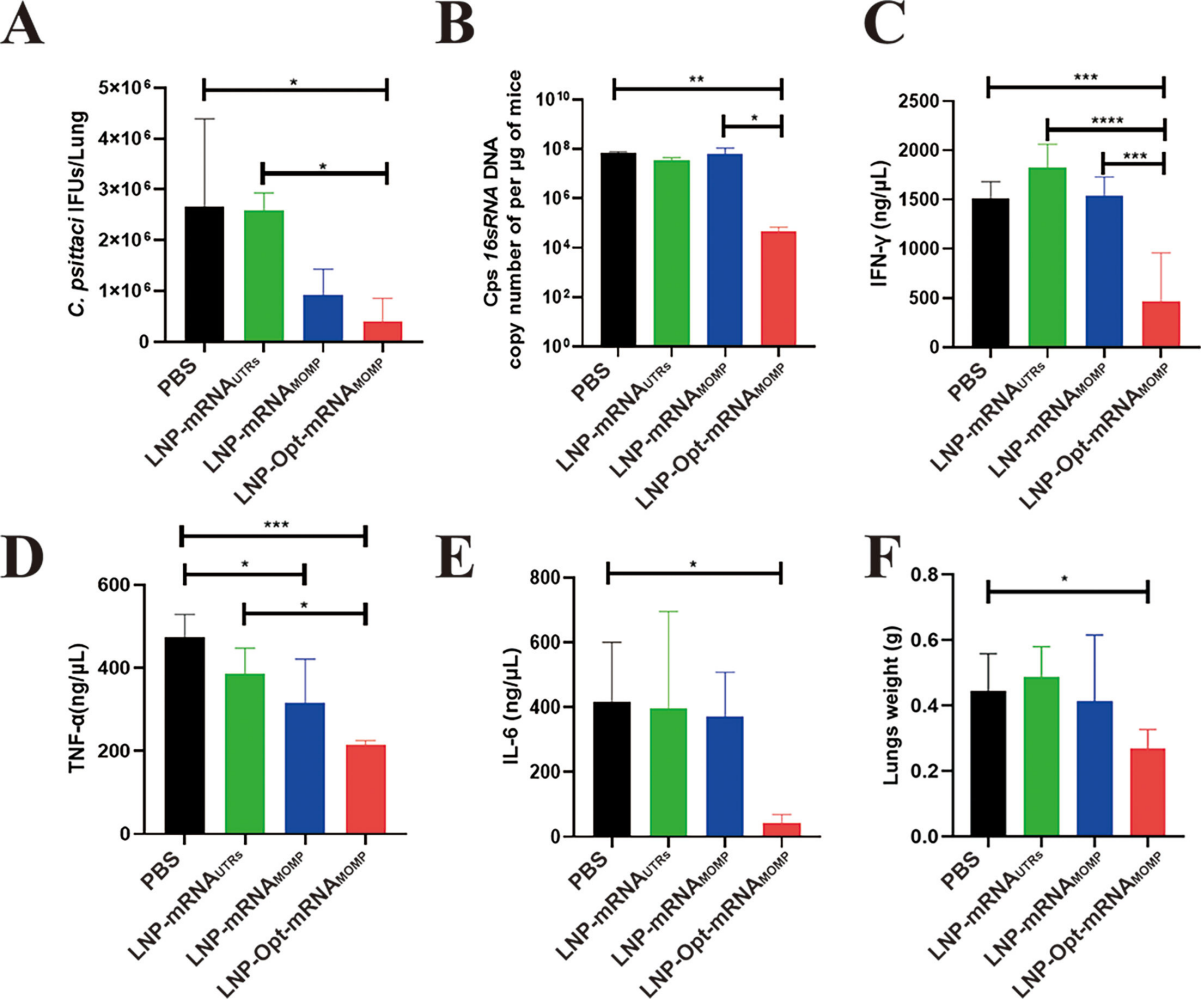
Evaluation of the protective effects targeting the lung tissue of mice after the challenge. (**A**) Detection of *C. psittaci* load in the lungs by immunofluorescence assay. (**B**) Determination of *C. psittaci* load in the lungs by qPCR assay. Cytokine IFN-γ (**C**), TNF-α (**D**), and IL-6 (**E**) levels in the lungs from *C. psittaci*-infected mice of each group. (**F**) Lung tissue weight analysis. Data in all groups were expressed with mean ± SD and analyzed by the one-way ANOVA (*P < 0.05, **P < 0.01, ***P < 0.001, and ****P < 0.0001).

On the 10th day after infection, mice from all the groups were euthanized, and their lung tissues were collected aseptically. IFA was conducted to quantify the presence of *C. psittaci* in lung tissues, as illustrated in Fig. 6A, the LNP-Opt-mRNA_MOMP_ group showed the lowest *C. psittaci* load in lung tissues, differing from both the PBS and LNP-mRNA_UTRs_ groups. qPCR amplification of the *C. psittaci* 6BC *16S rRNA* gene indicated that the *C. psittaci* load in lung tissues was significantly lower in the LNP-Opt-mRNA_MOMP_ group compared to both the PBS and LNP-mRNA_MOMP_ groups, as illustrated in Fig. 6B. These results indicate that LNP-Opt-mRNA_MOMP_ possesses effective efficacy in clearing *C. psittaci* in the lungs.

### The LNP-Opt-mRNA_MOMP_ group modulated the production of cytokines in BALB/c mice

Cytokine levels (IFN-γ, TNF-α, IFN-α, IL-2, IL-6, and IL-10) in lung tissue homogenate supernatant were quantified using ELISA. As shown in Fig. 6C, the LNP-Opt-mRNA_MOMP_ group showed the lowest IFN-γ levels, significantly differing from the PBS, LNP-mRNA_UTRs_, and LNP-mRNA_MOMP_ groups.

As illustrated in Fig. 6D, it is important to highlight that the LNP-Opt-mRNA_MOMP_ group exhibited the lowest TNF-α levels, significantly differing from both the PBS and LNP-mRNA_UTRs_ groups. No significant difference was observed between the LNP-Opt-mRNA_MOMP_ group and the LNP-mRNA_MOMP_ group. Additionally, the LNP-mRNA_MOMP_ group showed a significant difference compared to the PBS group.

Furthermore, as shown in Fig. 6E, the LNP-Opt-mRNA_MOMP_ group exhibited significantly lower IL-6 levels compared to the PBS group. No significant differences were found between the LNP-mRNA_UTRs_ and LNP-mRNA_MOMP_ groups when compared to the PBS group. No significant differences were observed among IL-2, IFN-α, and IL-10 (Fig. S2).

### The LNP-Opt-mRNA_MOMP_ group alleviated lung tissue damage and lesions in BALB/c mice

**Fig 7:**
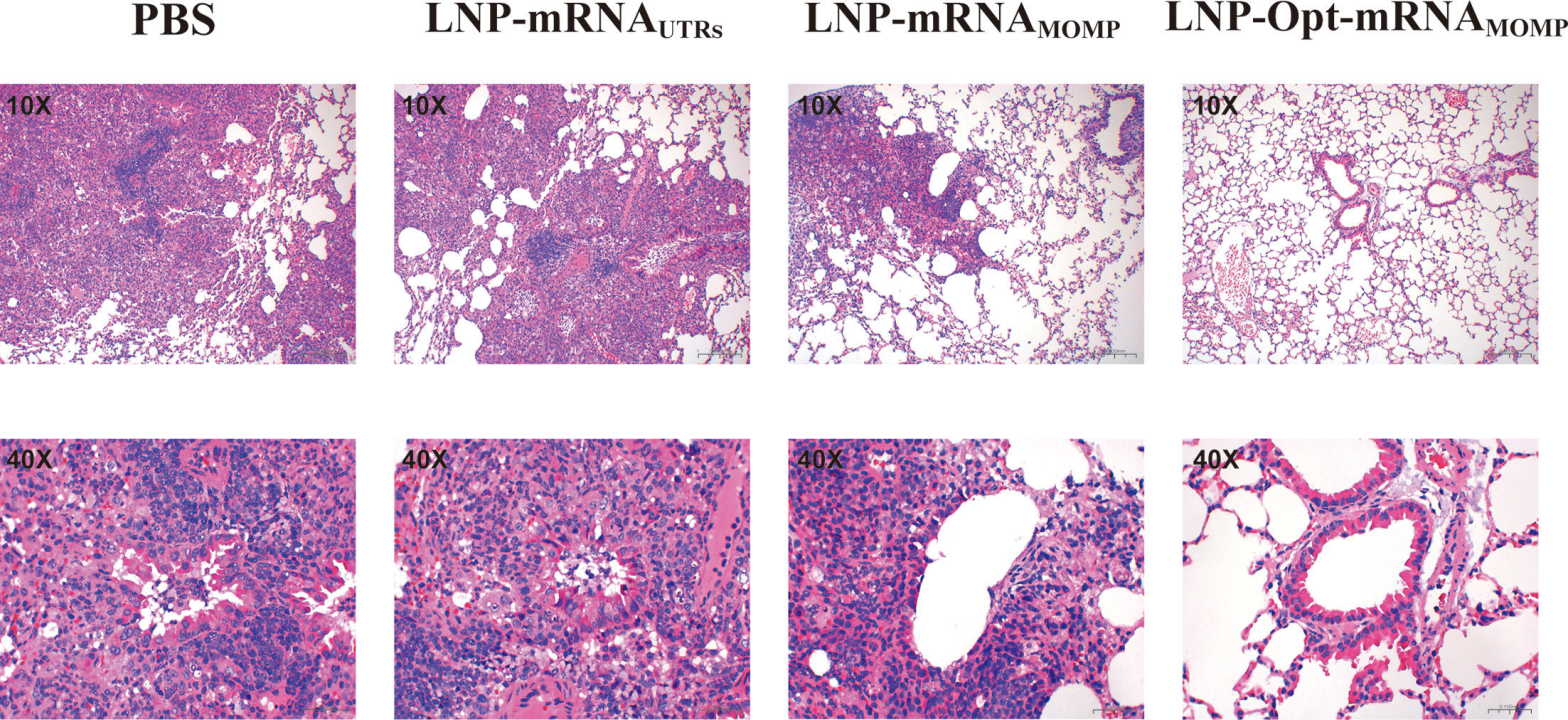
Representative results of H&E staining in the lungs of control and immunized mice 10 days after infection with *C. psittaci*. The alveolar structure of lung tissue in mice from the PBS and LNP-mRNA_UTRs_ groups exhibited damage characterized by significant infiltration of inflammatory cells inside the stroma. In the LNP-mRNA_MOMP_ group and the LNP-Opt-mRNA_MOMP_ group, the alveolar walls were slender and undamaged, exhibiting no notable pathological alterations.

We assessed lung tissue quality and conducted H&E staining on lung slices to further analyze tissue damage and lesion characteristics. The LNP-Opt-mRNA_MOMP_ group exhibited the lowest lung tissue weight, revealing a considerable disparity compared to the PBS group (Fig. 6F). No significant differences were found between the LNP-mRNA_UTRs_ and the LNP-mRNA_MOMP_ groups when compared to the PBS group. In the H&E staining of lung tissue (Fig. 7), the LNP-Opt-mRNA_MOMP_ group exhibited the lowest degree of pathological changes in lung tissue, with no apparent edema or inflammatory reactions. It showed a significant difference compared to the PBS group. No significant differences were found between the LNP-mRNA_UTRs_ and LNP-mRNA_MOMP_ groups when compared to the PBS group.

## DISCUSSION

Psittacosis is a zoonotic infection caused by *C. psittaci*, primarily presenting in humans as community-acquired pneumonia. Inaccurate or delayed diagnosis and treatment may lead to severe pneumonia, acute respiratory distress syndrome, multiple organ dysfunction syndrome, rapid patient decline, and potentially fatality (7, 38–40). The occurrence of psittacosis is typically sporadic, but there may also be a risk of epidemic outbreaks of infection (41–46). It is noteworthy that close contact with sick birds is commonly considered the source of psittacosis in humans, as evidenced by the scientific literature (47, 48). Recent research suggests that human-to-human transmission of psittacosis is an increasing public health threat (49–51). Considering the biosecurity threat of *C. psittaci*, public health authorities should enhance awareness of related infectious diseases like psittacosis and implement specific diagnostic methods and preventive strategies for high-risk groups. mRNA vaccines present a promising approach for the prevention and management of infectious diseases owing to their expedited production cycle, swift mass manufacturing capabilities, and elevated safety profile (16, 52–55). Developing safe and effective *C. psittaci* mRNA vaccines is crucial for preventing the spread and outbreak of related infectious diseases.

Since the 1990s, mRNA has been employed in vaccine research, but its instability and immunogenicity have slowed its progress. Excess immunogenicity can cause a severe immune response and inflammation. Clearing *Chlamydia*, regulating infection, and reducing pathological damage require proper inflammation. In contrast, excessive inflammatory cell cytokine production can promote infection and worsen immunological damage (56). The experiment improved the stability of the target nucleic acid sequences, mRNA_MOMP_ and Opt-mRNA_MOMP_, by integrating structural elements like the 5′-Cap, 3′-Poly(A) tail, and 5' and 3′-UTRs (57). Moreover, experimental identification revealed that transfected HeLa cells successfully translated and expressed the target antigen, MOMP protein, after LNP-mediated transfection of mRNA_MOMP_ and Opt-mRNA_MOMP_. Prior to *in vivo* evaluation, we assessed the cytocompatibility of our LNPs to define a safe therapeutic window for subsequent experiments. A concentration-dependent reduction in cell viability was observed, which is a common and well-characterized feature of cationic lipid-based nanoparticles. This cytotoxicity is primarily attributed to the positive surface charge of DOTAP, which can disrupt cell membrane integrity at higher concentrations (58). It is important to note that this represents a universal challenge in nanomedicine, and even clinically approved LNP formulations exhibit this property *in vitro*. The key is to identify a concentration that balances transfection efficiency with acceptable cytotoxicity. Our data clearly established that concentrations ≤ 15 nmol/10⁴ cells maintained high cell viability (>75%), and all subsequent functional experiments were conducted within this safe range. Due to the poor effect of LNP-mRNA_MOMP_ in mouse experiments, we optimized the nucleotide sequence based on the latest research progress and designed the LNP-Opt-mRNA_MOMP_ group to conduct the mouse experiments again, and the results were consistent with the expectations (13, 59–63). Immunizing BALB/c mice with LNP-Opt-mRNA_MOMP_ conferred immune protection against chlamydial infection. The specific relationship between the immunogenicity of LNP-Opt-mRNA_MOMP_ itself, related inflammatory responses, and immune protection against infection has yet to be fully understood in this experiment. Further in-depth exploration and research are required.

Research indicates that substituting natural nucleotides with modified versions, such as N^1^-methyladenosine or N^6^-methyladenosine for adenosine, 5-methylcytidine for cytidine, and 5-methoxyuridine, N^1^-methyl-pseudouridine, or pseudouridine for uridine, can significantly lower mRNA immunogenicity while improving the translation and expression efficiency of antigenic proteins (64–67). Consequently, research is focused on mRNA stability and improving delivery techniques to optimize cellular uptake and antigen presentation (68, 69). To prevent degradation of mRNA by nucleases, LNP encapsulation can be utilized to enhance mRNA stability and facilitate efficient cellular uptake (70–77). Producing a recombinant MOMP protein with correct conformational immunogenicity remains a considerable challenge; however, this hurdle is inherently overcome by the mRNA platform by leveraging the host’s cellular machinery for native antigen production. In this experiment, LNP can tightly bind to the mRNA_MOMP_ and Opt-mRNA_MOMP_. Transfection of LNP-encapsulated mRNA_MOMP_ and Opt-mRNA_MOMP_ into HeLa cells facilitates the translation and expression of MOMP antigenic protein in the cytoplasm (78, 79). Furthermore, studies have also demonstrated that LNP exhibits certain adjuvant properties, which, upon delivery *in vivo*, can induce relevant immune response reactions (80). A recent study published in the literature indicated that in rodent and non-human primate models, LNP can augment the immunogenicity of a tetravalent subunit dengue fever vaccine, eliciting strong specific humoral immune responses and T cell immunological responses (81). Similarly, Li et al. (82) demonstrated that LNP, when utilized as a vaccine adjuvant, can upregulate numerous genes linked to innate immune responses and viral infections, similar to those activated by viral infection. Additionally, it can improve both humoral and cellular immune responses induced by the vaccine, with a primary impact on CD4^+^ T cell responses. The aim was to provide a theoretical and practical basis for developing mRNA vaccines against *C. psittaci*. Despite one mouse dying in each of the LNP-mRNA_UTRs_ and LNP-Opt-mRNA_MOMP_ groups, this may have been attributable to individual variances among the mice. Flow cytometry analysis prior to pathogen challenge still indicates that LNP-Opt-mRNA_MOMP_ elicits robust cellular and humoral immune responses in mice, predominantly involving CD3^+^CD4^+^ cells. Research indicates that CD4^+^ T cells are essential in expediting *Chlamydia* clearance and preventing reinfection, playing a pivotal role during *Chlamydia* infection (83, 84). Additional investigations employing T cell and transcriptome analysis are essential to enhance comprehension and assessment of the distinct immunomodulatory effects of LNP.

Variations in antigen recognition pathways can cause CD4^+^ T cells to differentiate into specific subsets, resulting in the production of diverse cytokines (85). These cytokines have distinct functions in host defense against infections. Research shows that Th1 cells produce significant levels of pro-inflammatory cytokines, notably IFN-γ, aiding in the elimination of intracellular pathogens like viruses and chlamydia infections (86–89). Stimulation of the IL-12 pathway leads to IFN-γ-mediated tryptophan depletion via the indoleamine 2,3-dioxygenase pathway, which regulates *Chlamydia*’s growth cycle (90). Moreover, IFN-γ mitigates inflammation at infection sites by suppressing Th2 immune responses. The inability to downregulate Th2 immune responses may result in negative feedback, consequently worsening the depletion of IFN-γ. The experiment revealed that on the 10th day post-infection, the LNP-Opt-mRNA_MOMP_ group displayed markedly lower lung concentrations of IFN-γ and IL-6 in comparison to the PBS group. Notably, the *C. psittaci* load in the lungs of the LNP-Opt-mRNA_MOMP_ group was significantly reduced compared to the PBS group. The LNP-Opt-mRNA_MOMP_ group may elicit a Th1-Th2 immunological response to eradicate *Chlamydia*. Additional investigation is required to ascertain the mechanisms. Th17, Th22, and Th9 cells are present; however, their functions in *Chlamydia* infection remain unclear and require additional investigation.

### Conclusion

Expanding the selection of animal models could enhance the assessment of the immunoprotective efficacy of *C. psittaci* mRNA vaccines. In conclusion, LNP-based mRNA vaccines demonstrate potential for treating and preventing *C. psittaci* and are anticipated to be a promising strategy. This study provides significant insights into the capacity of LNP-Opt-mRNA_MOMP_ to elicit protective immunity against chlamydial infections. Future research should explore the potential of co-delivering two or more independent antigen-coding mRNAs to enhance and broaden immune responses.

This research aims to provide a theoretical basis and practical insights for developing *C. psittaci* vaccines based on MOMP, while also offering new perspectives for preventing and controlling other *Chlamydia* species and respiratory pathogens. Furthermore, future studies should aim to evaluate the vaccine’s efficacy against a diverse panel of *C. psittaci* isolates to explicitly validate its breadth of protection against natural genetic variation.

## Data Availability

The data used to support the findings of this study are included in the article.

## SUPPLEMENTAL MATERIAL

Fig. S1 spectrum.01438-25-s0001.pdf MOMP DNA template and UTR sequences.

Fig. S2 spectrum.01438-25-s0002.tif Cytokine IL-2, IFN-α, and IL-10 levels in the lungs of *C. psittaci*-infected mice from each group.

Supplemental legends spectrum.01438-25-s0003.docx Legends for Fig. S1 and S2.

ASM does not own the copyrights to Supplemental Material that may be linked to, or accessed through, an article. The authors have granted ASM a non-exclusive, world-wide license to publish the Supplemental Material files. Please contact the corresponding author directly for reuse.
