# Supplementary material for: Lipid nanoparticle-delivered mRNA vaccine encoding the MOMP of Chlamydia psittaci elicits protective immune responses in BALB/c mice
Source: Microbiol Spectr. 2025 Nov 5;13(12):e01438-25. doi: 10.1128/spectrum.01438-25 (PMC12671227; doi:10.1128/spectrum.01438-25)
Supplement: Fig. S1 — MOMP DNA template and UTR sequences. [file spectrum.01438-25-s0001.pdf]

Supplementary materials

S1

A. The part marked in yellow is the *MOMP* DNA template sequence:

1 ttacactctt ctacgagggt aattccaact tattctaagt ggcataagaa ataaaaatgt
61 gtacaaaaat ctgatagctc ttttattagc aagtataagg agttattgct tgaaatctat
121 gcctgaaaac agtctttttt cttatcgtct ttactataat aagaaaagtt tgttatgttt
181 tcgaataatg aactgtatgt tcatgcttaa ggctgttttc acttgcaaga cactcctcaa
241 agccattaat tgcctacagg atatcttgtc tggctttaac ttggacgtgg tgccgccaga
301 agagcaaatt agaatagcga gcacaaaaag aaaagatact aagcataatc tttagaggtg
361 agtatgaaaa aactcttgaa atcggcatta ttgtttgccg ctacgggttc cgctctctcc
421 ttacaagcct tgcctgtagg gaacccagct gaaccaagtt tattaatcga tggcactatg
481 tgggaaggtg cttcaggaga tccttgcgat ccttgcgcta cttggtgtga cgccattagc
541 atccgcgcag gatactacgg agattatgtt ttcgatcgtg tattaaaagt tgatgtgaat
601 aaaactttta gcggcatggc tgcaactcct acgcaggcta caggtaacgc aagtaatact
661 aatcagccag aagcaaatgg cagaccgaac atcgcttacg gaaggcatat gcaagatgca
721 gagtggtttt caaatgcagc cttcctagcc ttaaacattt gggatcgctt cgacattttc
781 tgcaccttag gggcatccaa tggatacttc aaagcaagtt cggctgcatt caacttggtt
841 gggttaatag ggttttcagc tgcaagctca atctctaccg atcttccaat gcaacttcct
901 aacgtaggca ttacccaagg tgttgtggaa ttttatacag acacatcatt ttcttggagc
961 gtaggtgcac gtggagcttt atgggaatgt ggttgtgcaa ctttaggagc tgagttccaa
1021 tacgctcaat ctaatcctaa gattgaaatg ctcaacgtca cttcaagccc agcacaattt
1081 gtgattcaca aaccaagagg ctataaagga gctagctcga attttccttt acctataacg
1141 gctggaacaa cagaagctac agacaccaaa tcagctacaa ttaaatacca tgaatggcaa
1201 gtaggcctcg ccctgtctta cagattgaat atgcttgttc catatattgg cgtaaactgg
1261 tcaagagcaa cttttgatgc tgatactatc cgcattgctc aacctaaatt aaaatcggag
1321 attcttaaca ttactacatg gaacccaagc cttataggat caaccactgc tttgcccaat
1381 aatagtggta aggatgttct atctgatgtc ttgcaaattg cttcgattca gatcaacaaa
1441 atgaagtcta gaaaagcttg tggtgtagct gttggtgcaa cgttaatcga cgctgacaaa
1501 tggtcaatca ctggtgaagc acgcttaatc aatgaaagag ctgctcacat gaatgctcaa
1561 ttcagattct aaggatttag tttatactat cctaactttt taaaccgcta tcagaacctg
1621 ggagtctccg ggttctgatt ttttaaatac cacccttttc

B. UTRs sequence

UTRs	Sequence
5’-UTR (β-globin-2)	AGAGCGGCCGCTTTTTCAGCAAGATTAAGCCCAGGGCAGAGCCATCT ATTGCTTACATTTGCTTCTGACACAACTGTGTTCACTAGCAACCTCAAA CAGACACC
3’-UTR (2β-globin)	AGCTCGCTTTCTTGCTGTCCAATTTCTATTAAAGGTTCCTTTGTTCCCTAA GTCCAACTACTAAACTGGGGGATATTATGAAGGGCCTTGAGCATCTGGA TTCTGCCTAATAAAAAACATTTATTTTCATTGCAGCTCGCTTTCTTGCTGT CCAATTTCTATTAAAGGTTCCTTTGTTCCCTAAGTCCAACTACTAAACTG GGGGATATTATGAAGGGCCTTGAGCATCTGGATTCTGCCTAATAAAAAA CATTTATTTTCATTGC
