# Supplementary figures and images for: Lipid nanoparticle-delivered mRNA vaccine encoding the MOMP of Chlamydia psittaci elicits protective immune responses in BALB/c mice
Source: Microbiol Spectr. 2025 Nov 5;13(12):e01438-25. doi: 10.1128/spectrum.01438-25 (PMC12671227; doi:10.1128/spectrum.01438-25)

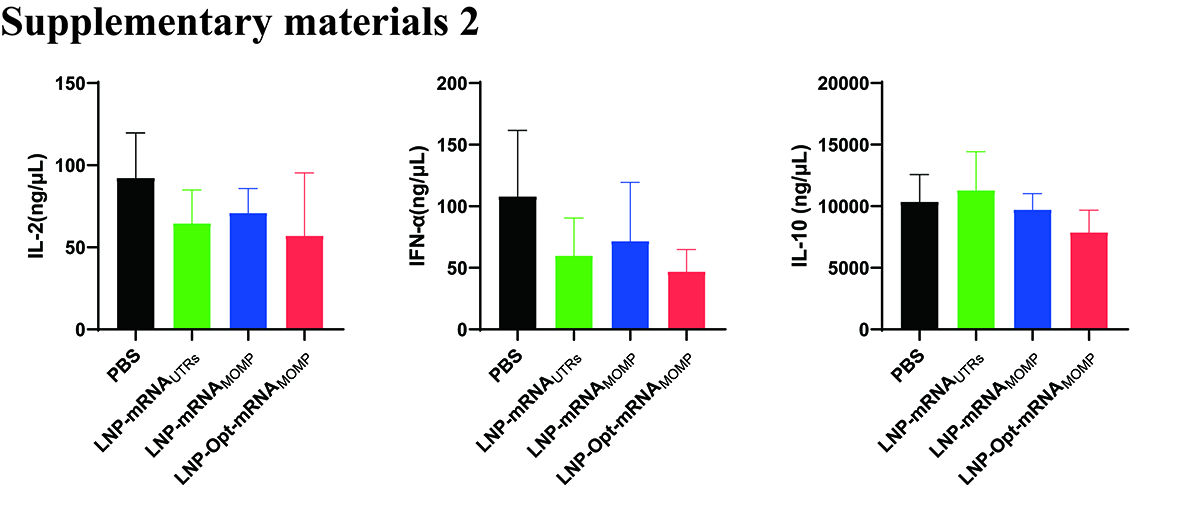

Supplement: Fig. S2 — Cytokine IL-2, IFN-α, and IL-10 levels in the lungs of C. psittaci-infected mice from each group. [file spectrum.01438-25-s0002.tif]
