# Supplementary material for: Lipid nanoparticle-delivered mRNA vaccine encoding the MOMP of Chlamydia psittaci elicits protective immune responses in BALB/c mice
Source: Microbiol Spectr. 2025 Nov 5;13(12):e01438-25. doi: 10.1128/spectrum.01438-25 (PMC12671227; doi:10.1128/spectrum.01438-25)
Supplement: Supplemental legends — Legends for Fig. S1 and S2. [file spectrum.01438-25-s0003.docx]

Supplemental legends

Supplemental legand 1: The part marked in yellow is the MOMP DNA template sequence (A); UTRs sequence (B).

Supplemental legand 2: Cytokine IL-2 , IFN-α , IL-10 levels in the lungs from *C. psittaci* infected mice of each group.
